# Artificial intelligence for understanding concussion: Retrospective cluster analysis on the balance and vestibular diagnostic data of concussion patients

**DOI:** 10.1371/journal.pone.0214525

**Published:** 2019-04-02

**Authors:** Rosa M. S. Visscher, Nina Feddermann-Demont, Fausto Romano, Dominik Straumann, Giovanni Bertolini

**Affiliations:** 1 Institute for Biomechanics, ETH Zurich, Zurich, Switzerland; 2 Department of Neurology, Interdisciplinary Center for Vertigo and Neurological Visual Disorders, University Hospital Zurich, University of Zurich, Zurich, Switzerland; 3 Swiss Concussion Center, Schulthess Clinic, Zurich, Switzerland; Julius-Maximilians-Universitat Wurzburg, GERMANY

## Abstract

**Objectives:**

We propose a bottom-up, machine-learning approach, for the objective vestibular and balance diagnostic data of concussion patients, to provide insight into the differences in patients’ phenotypes, independent of existing diagnoses (unsupervised learning).

**Methods:**

Diagnostic data from a battery of validated balance and vestibular assessments were extracted from the database of the Swiss Concussion Center. The desired number of clusters within the patient database was estimated using Calinski-Harabasz criteria. Complex (self-organizing map, SOM) and standard (k-means) clustering tools were used, and the formed clusters were compared.

**Results:**

A total of 96 patients (81.3% male, age (median [IQR]): 25.0[10.8]) who were expected to suffer from sports-related concussion or post-concussive syndrome (52[140] days between diagnostic testing and the concussive episode) were included. The cluster evaluation indicated dividing the data into two groups. Only the SOM gave a stable clustering outcome, dividing the patients in group-1 (n = 38) and group-2 (n = 58). A large significant difference was found for the caloric summary score for the maximal speed of the slow phase, where group-1 scored 30.7% lower than group-2 (27.6[18.2] vs. 51.0[31.0]). Group-1 also scored significantly lower on the sensory organisation test composite score (69.0[22.3] vs. 79.0[10.5]) and higher on the visual acuity (-0.03[0.33] vs. -0.14[0.12]) and dynamic visual acuity (0.38[0.84] vs. 0.20[0.20]) tests. The importance of caloric, SOT and DVA, was supported by the PCA outcomes. Group-1 tended to report headaches, blurred vision and balance problems more frequently than group-2 (>10% difference).

**Conclusion:**

The SOM divided the data into one group with prominent vestibular disorders and another with no clear vestibular or balance problems, suggesting that artificial intelligence might help improve the diagnostic process.

## Introduction

Concussion is often referred to as representing immediate and transient symptoms of mild traumatic brain injury; however, to date, no validated criteria exist to define concussion [1–4]. Clinical management of concussion is therefore a great challenge. Concussion reflects in a variety of affected functions and has a high complexity of symptom presentation with alteration of clinically observable parameters spread over a variety of domains ranging from cognition to balance or sleep. An increasing amount of evidence suggests that early and case-specific treatment is key to allow fast return to daily life [5, 6]. Of note, although it is broadly acknowledged that concussion is a multi-dimensional problem [4], most research has focused on imaging, neuropsychological and symptom testing [4, 7–9]. While there has been an increasing amount of studies looking into vestibular impairments in concussion patients in the last years [10–13], there is still a lack of understanding [14]. This lake of understanding might partly be due to the complexity of interpreting the overall result from a vestibular evaluation, which consists of multiple tests that each provide valuable information. However, a good understanding of the vestibular system is fundamental to determine the aetiology and provide treatment recommendations [4, 15, 16].

Artificial intelligence (AI) can be used as a tool to summarize multiple parameters and make interpretation of overall results easier. Specifically, machine learning (ML) has been used in an increasing amount of studies to improve clinical diagnoses and explore unexplained phenomena [17]. ML algorithms take huge numbers of parameters into account, beyond the scope of human capability, thereby increasing diagnostic speed, accuracy and reliability. This can lead to lower healthcare costs and increased patient satisfaction [17]. ML can also help identify which features or combination of features discriminate between multiple patient populations [18]. This information can be used to optimize diagnostic criteria and improve patient monitoring. In concussion research, ML has already successively been used on imaging [9, 19–22], neuropsychological [23, 24], eye movement [25], and clinical [26] data to improve the diagnostic process. It showed to be able to distinguish between concussed and control subjects [9, 20, 21, 25, 27]. However, to our knowledge ML has not been used before on a vestibular database of concussion patients.

Given the uncertainty in the current diagnostic process for concussion, an ML bottom-up approach that is independent of a specific diagnosis is proposed. The main objective was to evaluate if ML can be used on a relatively small vestibular database with little information to drive supervised learning. To perform this evaluation, the performances of a standard and a complex clustering tool were compared, and the percentage of overlap between the methods was calculated. In addition, there were two secondary objectives. First, we aimed to identify which features were considered most important by the ML algorithm for separating patients into different subgroups. Second, upon comparing the subgroups formed by the ML algorithm, we aimed to identify whether significant differences between subgroups indicate that they may correspond to separate phenotypes. The goal of this study was to explore the use of ML for novel insights into the differences in phenotypes between patients with concussions based on objective vestibular and balance performance. This study could be helpful in the future for improving assessment batteries and diagnostic criteria.

## Methods

In this retrospective study, a cluster analysis was conducted on the balance and vestibular diagnostic database of the Swiss Concussion Center (SCC). The study was approved by the cantonal ethical committee of Zurich (2017–01208).

### Database

The balance and vestibular diagnostic database consists of data from the following tests:

- Balance diagnostic testing: sensory organization test (SOT, from which 5 variables were extracted) [28, 29].
- Vestibular diagnostic testing: dynamic visual acuity (DVA, 8 variables) [30, 31], video head impulse test (V-HIT, 9 variables) [31–33], cervical and ocular vestibular-evoked myogenic potential (cVEMP, oVEMP, together 3 variables referred to as VEMPs) [31, 33], subjective visual vertical (SVV, 11 variables) [34], caloric (11 variables) [31, 33], and Fundus photography (3 variables) tests [35].

S1 Table provides an overview of the parameters included in the analysis for each test. Epidemiological data (sex, age, time between diagnostic testing and concussive episode) were also included in the analysis. Additionally, symptoms were recorded with a modified version of the concussion symptom inventory (CSI) [36] upon the first visit of the patient to the SCC. Patients had to fill in if the symptoms (Table 1) were absent or present.

**Table 1:** Epidemiological data for the included patients. IQR = interquartile range; SCC = Swiss Concussion Center.

	Male	Female
**Total [n (%)]**	78 (81.3)	18 (18.7)
**Age (years, median [IQR])**	25.0 [11.0]	25.0 [13.0]
**Most common time between injury and visit to SCC (%)**	≤ 2 weeks (15.4)	≤ 2 weeks (5.6)
	≤ 4 weeks (19.2)	≤ 4 weeks (16.7)
	≤ 3 months (34.6)	≤ 3 months (22.2)
	> 3 months (30.8)	> 3 months (55.6)
**Most common types of sports (%)**	Ice hockey (57.7)	Ski & Snowboard (27.8)
	Soccer (7.7)	Ice hockey (11.1)
	Handball (5.1)	Handball (11.1)
**Amnesia (%)**	Anterograde (23.4)	Retrograde (25.0)
	Retrograde (13.0)	Anterograde (9.1)
**Current symptoms reported during first visit to SCC (%)**	Headache (66.7)	Headache (62.5)
	Dizziness (47.0)	Dizziness (56.3)
	Neck pain (42.2)	Difficulty concentrating (37.5)
	Difficulty concentrating (39.4)	Blurred vision (37.5)
	Blurred vision (28.8)	Neck pain (18.8)
	Sensitivity to light (22.7)	Sensitivity to light (18.8)
	Balance problems (20.0)	Balance problems (18.8)
	Feeling confused (6.1)	Nausea (12.5)
	Coordination problems (4.5)	Difficulty remembering (6.3)
	Nausea (3.0)	Feeling confused (0.0)
	Difficulty remembering (0.0)	Coordination problems (0.0)
	Feeling slowed down (0.0)	Feeling slowed down (0.0)

#### Participants

The study population consisted of 212 patients suspected of suffering from a sports-related concussion (SRC) or post-concussive syndrome (PCS). SRC was defined as a traumatic brain injury induced by biomechanical forces as defined by a concussion in a sports group [1]. PCS was defined as still having concussive symptoms more than 14 days after a concussive episode. Only patients who performed balance and vestibular diagnostic testing in the SCC between January 2015 and November 2017 were included. All patient data were excluded from the analysis if one of the following exclusion criteria was met:

- the patient did not perform balance and/or vestibular tests,
- >5% of the patient’s data were missing from the database
- the patient did not allow the use of his/her data for research purposes

### Procedures

According to the in/exclusion criteria, patients were selected from the database of the SCC, and their data were imported into MATLAB (R2016b, The Matworks, USA) with an automated custom-designed routine. The imported data were stored into a single matrix, where each row represented one patient and each column one variable. Patient outcomes on the balance and vestibular tests and their epidemiological data were used for cluster analysis. In total, 53 variables were used for the cluster analyses, see S1 Table for more detailed description. Symptoms reported during the first visit to the centre were not used for the cluster analysis but were used for statistical analyses of the subgroups defined by the cluster analysis.

Two different clustering tools were used:

- K-means [37]
- Kohonen’s self-organizing map [38, 39] (SOM)

K-means was chosen because it is a standard clustering tool. It was implemented in MATLAB using the Statistics and Machine Learning Toolbox (R2016b, The MathWorks, USA). The cluster centroid positions were randomly initialized, and the squared Euclidean distance metric was used. The number of clusters was determined according to the mode of 500 repetitions of the cluster evaluation tool from the same toolbox using the Calinski-Harabasz (CH) [40], Silhouette (SI) [41], Gab [42], and Davies Bouldin (DB) [43]criteria.

SOM is an artificial neural network suitable for exploratory data mining, and outcomes can be used for data compression, pattern recognition, and diagnostic purposes [38, 44, 45]. The choice of SOM as clustering tool was motivated by its ability to deal with relatively small datasets and due to its visualization properties [44, 46]. For a more detailed description of the SOM algorithm used, see the SOM Toolbox for MATLAB 5 [39].

The size of the SOM was decided by minimizing the quantization and topographic errors [47, 48]. After the SOM was run on the data, the output (later on referred to as the first layer) was used as the input for another SOM (the second layer), and its size was forced to be smaller than the previous layer. New layers were progressively added until the desired number of data clusters was reached, as previously estimated using the cluster evaluation tool described above. Each layer was set to have a global sheet-shaped map and a local hexagonal lattice structure [39, 44, 46]. Initialization of the neuron’s weight was random and an ep neighbourhood function was used [39, 44, 49]. The maps were trained in two phases using the batch algorithm: 1) a rough training phase with a large initial neighbourhood width and a learning rate set at 20 epochs and 2) a fine-tuning phase with a small initial neighbourhood width and a learning rate set at 50 epochs [46].

To improve the stability of the cluster outcomes, SOM and k-means were both repeated 100 times, after which the mode was taken [50]. This procedure was repeated three times to evaluate the stability of the results. If less than 10% of the people were clustered differently across the three repetitions of the clustering procedure, the outcome was considered stable. From those three repetitions, the mode was taken for evaluation of the feature determining the clusters.

In the context of the first secondary aim, leave-one-test-out analyses were conducted to evaluate the dependency of the tool on a specific input. During the leave-one-test out analyses, each of the 8 tests was taken out once from the matrix used to train the network. The number of patients clustered differently when a test was removed was counted. These analyses were only conducted if the cluster outcomes were considered stable, according to the criteria described above. A further leave-one-variable-out analysis was calculated for the single variables of the tests (each of the 53 features was taken out once from the matrix to train the network and the number of patients clustered differently when a feature was removed was counted). The leave-one-variable-out analyses was only conducted if an entirely left out test, resulted in a different clustering of at least 20% of the patients during the leave-one-test-out analyses. A Principal Component Analyses (PCA) was also conducted, since it is often seen as the basis for multivariate analyses [51, 52]. PCA extracts the information needed to explain the highest amount of variance within a dataset and expresses this information as a set of new orthogonal variables called principal components (PCs) [51]. By comparing the formed PCs with the outcomes of the leave-one-out analyses, an indication can be made if the variables that influence the clustering outcomes most of all, are also the once explaining most of the variation in the dataset.

### Statistical analysis

Normality of data distribution was checked with the Kolmogorov-Smirnov test and QQ-plots. It was concluded that the data was relatively normally distributed. However, there were some outliers; since there were no good grounds for why these outliers should be removed, they were kept in. To give a good overview of the data, median and IQR values were reported. Mann-Whitney-U, chi-squared and Fisher’s exact tests were used to compare the subgroups. Eta squared (η^2^ = Z^2^/N) was used to calculate the effect sizes [53]. The significance was initially set at *α* = 0.05 but was corrected to *α* = 0.0006 after a Bonferroni correction [54].

Before cluster analyses were performed, missing data were replaced by the median scores (to minimize the influence of the outliers), and z-scores were calculated (to prevent variables with bigger ranges/values to have a higher influence on the machine learning outcomes) in MATLAB. Z-scores were used for all further analyses, except for CI, to make clinical interpretation possible. The complete analysis was once performed with and once without outliers, but outcomes where not much affected, therefore no further steps were taken. All statistical analyses were conducted in SPSS, and missing data was indicated by 9999 to enable processing of input data and listwise deletion.

## Results

### Participant characteristics

From the 212 patients in the database of the SCC, 121 performed balance and vestibular diagnostic testing, 25 of which had more than 5% missing data. In total, 96 patients were included in this study, leading to a matrix of 96 rows by 53 columns with 0.85% missing data for the cluster analyses. From the current symptoms reported by the patients, 17.29% of the data were missing, considering the 96 included patients. An epidemiological overview of the included patients is shown in Table 1.

### Outcomes of the clustering procedure

The mode of 500 repetitions of cluster evaluation showed the data could best be grouped into 2 groups, a value recommended in 94% using CH, 82% using SI. Gab and DB criteria did not have a mode that appeared >50% of the repetitions (for Gab criterion highest mode was for k = 5 in 25% of the cases, for DB the highest mode was 22 in 20% of the cases)

The k-means divided the data into 2 groups with 53 and 43 patients when taking the mode of the 3x100 repetitions. The result of the k-means was not considered stable since a number of patients ranging from 22–25 (i.e., >10%) were clustered differently when repeating the clustering procedure of 100 repetitions 3 times. Due to this instability, the feature and group analyses were not conducted for the k-means.

The optimal size for the SOM was identified to be 48 cells (8x6) in the first layer with a quantization error of 5.629 and a topographic error of 0.01. Five layers were required to achieve a separation in two clusters with 58 and 38 patients, respectively. The fifth layer had 4 cells (2x2), a quantization error of 0.318 and a topographic error of 0. The results of the SOM in layer 5 were considered stable (the number of patients changing groups ranged from 4 to 6, i.e., <10% were clustered differently when repeating the clustering procedure of 100 repetitions 3 times).

A separation into two cluster becomes clearly visible from layer 4 onwards. On the first layer no clear separation into two clusters was visible, from the 48 nodes available, patients were divided over 33 nodes. While the patients grouped in the nodes right above are relatively close together, patients grouped in the lower left corner were relatively far apart, as shown in the unified distance matrix (U-matrix) provided in S1 Fig.

**Fig 1:**
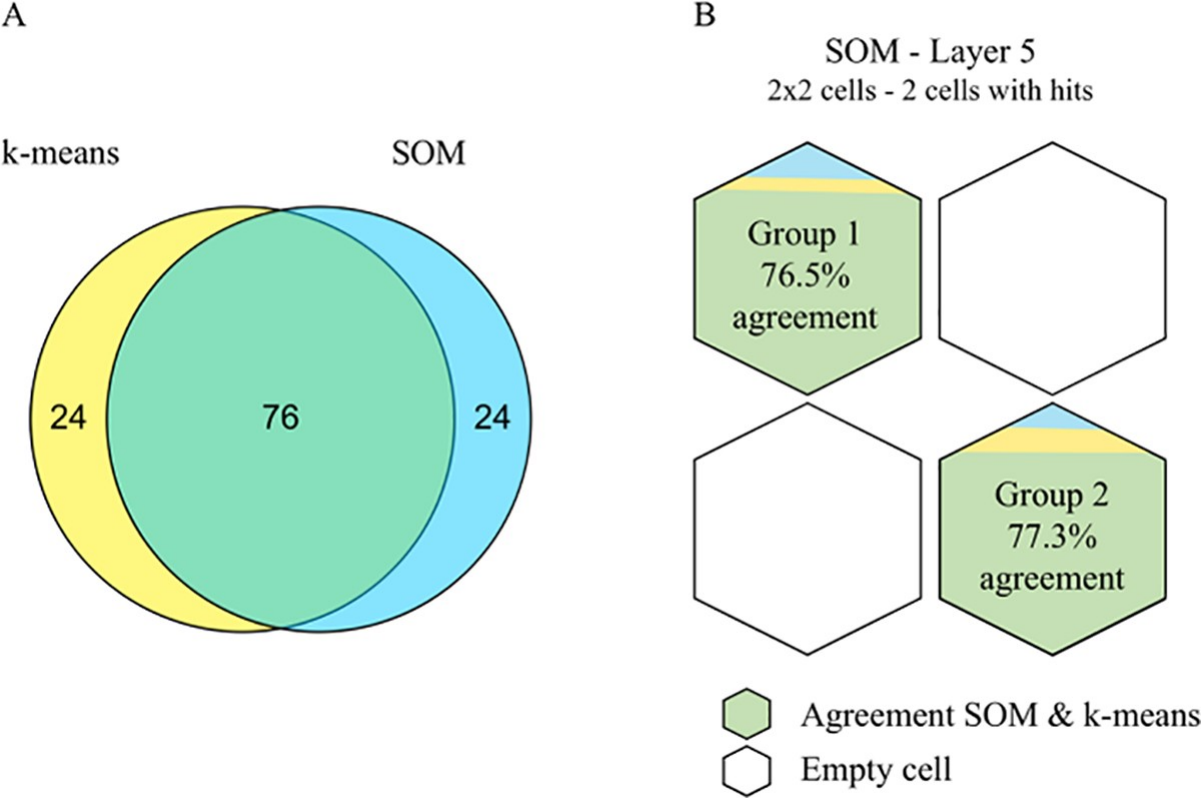
Comparison between clustering outcomes of the k-means and SOM. (A) Venn diagram of the overlap between the k-means and the SOM. (B) Overlap between the two clustering methods shown as the percentage of patients from each group identified by the SOM that was identically classified by the k-means. For visualization, the two groups are drawn on the map of layer 5 of the SOM.

The percentage of overlap between the clusters of the two methods was 76%, as shown in Fig 1A. This difference is caused by a change in classification of an equal number of patients from both groups (Fig 1B).

### Evaluation of the feature determining the clusters

The outcomes of the leave-one-test-out and leave-one-variable-out analyses are shown in Table 2. Removing the variables for the caloric or for the DVA resulted in larger changes in the patients’ classification (24%), suggesting that these two variables were the most important for the clustering algorithm. Lower percentages were observed for HIT, SOT, and Fundus (>10-<20%). The lowest number of classification changes was observed when excluding either VEMPs, SVV or epidemiological data (<10%). According to the leave-one-variable-out analyses, the clustering outcomes mostly depend on the variables describing the mSPV (19%) induced by the caloric test and the DVA LogMAR outcomes during the static and dynamic conditions (19% and 17%, respectively).

**Table 2:** Outcomes of the leave-one-variable-out and leave-one-test-out analyses to evaluate the dependency of the SOM on specific inputs. DVA = dynamic visual acuity; SOT = sensory organization test; V-HIT = video head impulse test; General = sex/age/time since concussive incident/sport type; SVV = subjective visual vertical; VEMPs = cervical and ocular vestibular-evoked myogenic potential; mSPV = maximal speed of the slow phase velocity.

Leave-one-test-out		Leave-one-variable-out	
Without the following test	Clustered differently [%]	Without the following variable	Clustered differently [%]
Caloric	24	mSPV-44	19
		mSPV-sum	17
		mSPV-30	17
		Index-30	11
		Index-44	9
		Difference	8
		Dominancy	5
DVA	24	Static	19
		Dynamic	17
		Loss	14
		Velocity	6
SOT	15		
Fundus	13		
V-HIT	13		
General	10		
SVV	9		
VEMPs	7		

The PCA outcomes can be found in S2 Table. PC1 explained 12.7% of the observed variation. After rotation, PC1 depended completely on HIT outcomes, PC2 on Caloric mSPV outcomes, PC3 on SOT outcomes, PC4 on DVA static/dynamic outcomes, see S2 Table. Together PC1 up and until PC4 explained 38.6% of the variance. 16 PCs were needed to explain >80% of variance.

### Evaluation of the two clusters of patients

Group-1 scored significantly lower on the SOT-cs (p = 0.0004) score and on the caloric mSPV-30/44/sum outcomes (p = 0.000009, p = 0.00009, and p = 0.00000006, respectively), and had increased scores for the DVA under static and dynamic conditions (p = 0.00001 and p = 0.0002, respectively); see Table 3.

**Table 3:** Confidence intervals per test for each subgroup and the effect sizes of the difference between the subgroups.

Sig. different variables*	Group 1	Group 2	Effect size
	Median[IQR]	Median[IQR]	Eta squared
**SOT-cs**	↓ 69.00 [22.30]	↑ 79.00 [10.50]	13.2%^†^
**DVA-dynamic**	↑ 0.38 [0.84]	↓ 0.20 [0.20]	18.6%^†^
**DVA-static**	↑ -0.03 [0.33]	↓-0.14 [0.12]	19.5%^†^
**Caloric mSPV-30**	↓ 7.25 [6.70]	↑ -13.30 [10.00]	19.5%^†^
**Caloric mSPV-44**	↓ 7.30 [6.20]	↑12.35 [9.50]	25.3%^†^
**Caloric mSPV-sum**	↓ 27.60 [18.20]	↑ 50.95 [31.00]	30.7%^‡^

*α<0.001 for all variables in the independent variables Mann-Whitney-U test on normalized data

IQR = interquartile range; SOT-cs = sensory organization test composite score; DVA = dynamic visual acuity measured as log of the minimum angle resolvable; mSPV = maximal speed of the slow phase velocity measured in degrees per second; 30 and 44 refer to the temperature of the water used; sum = summary score calculated by caloric software; Effect size for eta squared:

^†^ >0.1 for a small effect

^‡^>0.3 for a medium effect, and >0.5 for a large effect.[53].

Group-1 also scores significantly lower on the PC2 (p = 0.0000004) and had increased scores for PC-4 (p = 0.0002). A scatter plot visualizing the data distribution over the PCs can be found in S2 Fig.

No significant differences between the groups were found in the symptoms reported by the patients. Nonetheless, group-1 evidenced the tendency to report more frequently headache, blurred vision and balance problems, while less anterograde amnesia (Table 4).

**Table 4:** Difference in frequency for current symptoms reported by patient.

More in Group 1	More in group 2	Similar in both groups
[≥10% diff]	[≥10% diff]	[<10% diff]
Headache	Anterograde Amnesia	Sensitivity to light
[76 vs. 59%]	[14 vs. 24%]	[27 vs. 18%]
Blurred vision		Dizziness
[39 vs. 24%]		[45 vs. 51%]
Balance problems		Difficulty concentrating
[27 vs. 15%]		[39 vs. 39%]

## Discussion

### Summary of main findings

This study explored the use of machine learning (ML) as a bottom-up approach to generate novel insight into differences in phenotypes between concussion patients based on their epidemiological, balance, and vestibular diagnostic outcomes. A standard clustering tool (k-means), which was tested on the same dataset as a reference for clustering performance, failed to achieve a stable result. The complex clustering tool (self-organizing map, SOM) identified two stable groups, the separation of which was dictated mainly by the parameters of the caloric maximal slow phase velocity (mSPV) and dynamic visual acuity (DVA) tests. Although no diagnosis was provided, the average values of the variables in the two groups suggested that the patients assigned by the ML algorithm to the two groups formed clusters that are clinically distinct: one group included patients with prominent vestibular disorders, and the other included those with no clear vestibular or balance problem.

### Relevance of the diagnostic tests

Observing how the ML algorithm structured the two clusters allowed us to indirectly infer the distinctive aspect of the phenotypes. According to the leave-one-test-out analysis, the definition of the clusters depended mainly on the availability of the outcomes of the caloric test and the DVA test. This implies that the two tests are important in the diagnostic process since if one of them would not have been performed, 24% of the patients would have been classified differently.

For the parameters of the caloric test, the mSPV appeared to have a prominent role. The mSPV indicates the overall ability of the vestibular system to respond to stimulation (irrigation of the ear with warm or cold water generates a powerful activation of the semicircular canals) and is also known as vestibular reflectivity [55]. Previous studies support that mSPV is important in the diagnosis and management of vertigo disorders [56, 57]. While mSPV is not often considered in concussion management, our results strengthen the need for including caloric testing in diagnostic test batteries for concussion. The relevance given by the ML algorithm to the DVA parameters, though important, is less surprising since previous research showed a deficit in DVA performance in a subgroup of concussion patients [10, 58].

The PCA outcomes support the conclusion drawn by the leave-one-test-out analyses, where PC2 (consisting of caloric mSPV) and PC4 (consisting of DVA static/dynamic) were shown to be significantly different between the two clusters. While PC1 explained most of the variation within the dataset, it did not discriminate between the two clusters, as is highlighted in S2 Fig.

Importantly, while the above reasoning provides clear evidence that the caloric and DVA tests need to be part of the diagnostic assessment of concussion patients, it does not imply that other tests can be discarded. PCA outcomes show that over 15 components are needed to explain 80% of the variation within the data. The strength of an ML algorithm is in combining all available inputs, and each test and each parameter within a test may provide fundamental information for specific cases.

### Clinical significance of the two clusters of patients

The two patient clusters identified by the ML algorithm appear to represent clinically distinct patient subgroups. The significantly lower mSPV observed for group-1 with respect to group-2 suggests a reduced vestibular function, a finding previously linked to self-reported postural unsteadiness [16]. This finding was further corroborated by a pathological SOT-cs that was significantly lower than for that of the group-2 patients, whose SOT-cs values were within the 95% of the normative population [59]. Accordingly, group-1 also scored worse on the DVA, which is in line with previous findings of Zhou and colleagues [10], and tended to report symptoms of balance problems and blurred vision more frequently compared to group-2. This last observation is of particular importance because symptoms were not provided to the algorithm, and the observed trend further corroborates the separation between the identified groups. Of note, the ML algorithm was not provided with diagnoses, and the patients were not selected accordingly. The ML algorithm therefore independently suggested the presence of a distinct subgroup with vestibular impairment within our patients, corroborating the hypothesis that patients with clear balance and vestibular pathology may form a particular subgroup within the concussive population [60, 61]. This study therefore demonstrates that patients within this subgroup, which until now, have been identified mostly based on symptoms [10, 60], can be recognized using objective variables and a bottom-up approach.

### Clinical innovation

This study introduced the use of a multi-layer self-organizing map (SOM) to gain insight into complex pathologies. By systematically analysing the outcomes of the SOM with leave-one-parameter-out analysis, knowledge about how the clusters are generated can be extracted and used to develop a novel view of the complexity of multi-dimensional pathologies.

### Limitations & remarks

The number of patients included in the analysis was relatively low, both for an ML study and with respect to the number of features. This limiting factor may explain, for example, the instability of the k-means outcomes, reducing the value of the comparison between the clustering methods. It might be possible to stabilize the k-means outcomes by performing PCA before, and perform the cluster analyses on the formed PCs [62]. The SOM, however, did give a stable outcome that can be considered reliable and is in line with previous works, supporting the use of SOM for relative small databases [44]. Due to the limited number of patients, however, the database does not represent the overall population of concussion patients, which should be kept in mind when interpreting the results of this study. Since 81% of the included patients were male, 85% were under 35 years old and 85% were (professional) athletes, the obtained results are specifically relevant for the management of sports-related concussions (SRC) [63].

The time delay between the concussive episode and the diagnostic battery varied considerably within the database. This is an important factor to take into account since previous studies showed that outcomes of balance and vestibular tests depend on the amount of days after the concussive episode [1]. The parameter ‘time delay (in days)’ was provided as an input for the ML algorithm to enable the algorithm to account for this effect, if present in our data, by “learning” it.

Instead of visualizing the data by using PCs from the PCA, other techniques such as t-distributed stochastic neighbour embedding technique (t-SNE) [64] or Uniform Manifold Approximation Projection (UMAP) [65] could also have been used. T-SNE has been increasingly used in the last years, and has shown promising outcomes, also in the field of concussion research[66], however large datasets are needed to get stable outcomes. UMAP was developed in 2018, and showed to be faster compared to t-SNE, provided better scaling and it showed to be better suited for small datasets [65]. It would therefore be interesting to explore concussion data with UMAP. The choose for using PCA instead of UMAP in this study was motivated by the fact that clinicians are more familiar with the PCA, therefore it was seen as a better fit for our target audience.

While this study compared SOM with k-means, it would also have been possible to compare the SOM outcomes with more basic approaches, such as factor analysis (FA). FA is a statistical method to investigate the relationship between items in a dataset. By combining explorative and confirmatory FA clinically relevant factors might be identified [67]. However, it is also known that FA is a large-sample procedure; generalizable or replicable results are unlikely if the sample is too small [68]. It was therefore decided that FA was not a good fit for the investigated dataset.

### Recommendations for future research

The clinical relevance of the already identified subgroups should be further evaluated by future studies with a focus on parameters, such as recovery time and response to specific treatment options. An extension of the ML algorithm to include non-vestibular and non-balance parameters (e.g., previous concussions, neuropsychological outcomes, and cervical spine evaluation) is also needed to validate the current subgroups in a more complex diagnostic context and to identify others.

## Conclusion

Overall, this exploratory retrospective study introduced a novel tool that blindly identified a patient subgroup of those with prominent vestibular disorders within a population of patients with concussions. This tool uses an unsupervised machine-learning algorithm (multi-layer self-organizing map) on balance and vestibular data. Caloric and DVA tests were shown to be most important for defining the two groups. Further research is necessary to examine the clinical implications of the subgroup with vestibular impairments.

## Supporting information

S1 Table Parameters used for cluster analyses. Details on the tests implementations can be found in the articles referenced in the method section.
(DOCX)

S2 Table PCA outcomes. (DOCX)

S1 Fig Unified distance matrix of the SOM. (TIF)

S2 Fig Scatter plots visualizing the distribution of the formed clusters over the principal components of the PCA. (A) 2D scatter plot: PC 1 plotted against PC 2. (B) 3D scatter plot: PC 2, 3, and 4. Yellow markers were clustered in group-1; Blue markers were clustered in group-2.
(TIF)
