# Supplementary material for: Artificial intelligence for understanding concussion: Retrospective cluster analysis on the balance and vestibular diagnostic data of concussion patients
Source: PLoS One. 2019 Apr 2;14(4):e0214525. doi: 10.1371/journal.pone.0214525 (PMC6445465; doi:10.1371/journal.pone.0214525)
Supplement: S1 Table — Details on the tests implementations can be found in the articles referenced in the method section. (DOCX) [file pone.0214525.s001.docx]

|  | |  | |
| --- | --- | --- | --- |
|  | |  | |
|  | |  | |
| S1 Table Parameters used for cluster analyses  Details on the tests implementations can be found in the articles referenced in the method section. | | | |
|  | |  | |
| Parameters used to perform clustering procedure | | | |
| Test | | **Parameter(s) included** | |
| Epidemiological data | |  |  |
|  | | Gender | 0 = male  1 = female |
|  |  | Age | (Years) |
|  |  | Time since injury | (Days) |
| Balance diagnostic testing |  | |  |
| Sensory Organization Test (SOT) | | Composite score  VEST  VIS  PERF  SOM | 0-100  0-100  0-100  0-100  0-100 |
| Vestibular diagnostic testing | |  |  |
| Dynamic Visual Acuity (DVA) | | Average speed Left  Static Left  Dynamic Left  Loss Left  Average speed Right  Static Right  Dynamic Right  Loss Right | (Degrees per second)  (LogMAR)  (LogMAR)  Dynamic-Static  (Degrees per second)  (LogMAR)  (LogMAR)  Dynamic-Static |
| Video Head Impulse Test (V-HIT) | | Gain posterior arc left  Gain posterior arc right  Gain anterior arc left  Gain anterior arc right  Gain horizontal arc left  Gain horizontal arc right  Asymmetry anterior  Asymmetry lateral  Asymmetry posterior | Average value  Average value  Average value  Average value  Average value  Average value  (Highest value – lowest)/Highest*100%  (Highest value – lowest)/Highest*100%  (Highest value – lowest)/Highest*100% |
| Vestibular Evoked Myogenic Potential (VEMP)  Cervical VEMP (cVEMP)  Ocular VEMP (oVEMP) | | Threshold  Asymmetry  Asymmetry | Till which value a response is present (90/95/100 dB)  (LA-SA)/(R+L)*100%  (LA-SA)/(R+L)*100% |
| Subjective visual vertical (SVV) | | Head straight end value  Head straight head tilt  Head left end value  Head left head tilt  Head right end value  Head right head tilt  Head straight SD  Head left SD  Head right SD  Overall SD  Overall end value | (Degrees)  (Degrees)  (Degrees)  (Degrees)  (Degrees)  (Degrees)  (Degrees)  (Degrees)  (Degrees)  (Degrees)  (Degrees) |
| Caloric | | Maximal speed of the slow phase velocity right 30°C  Fix Index right 30°C  Maximal speed of the slow phase velocity left 30°C  Fix Index left 30°C  Maximal speed of the slow phase velocity right 44°C  Fix Index right 44°C  Maximal speed of the slow phase velocity left 44°C  Fix Index left 44°C  Side difference  Directional preponderance | (°/s)  (%)  (°/s)  (%)  (°/s)  (%)  (°/s)  (%)  (%)  (%) |
| Fundus photography | | Angle left eye  Angle right eye  Difference | (Degrees)  (Degrees)  (Angle Left + Angle right)/2 |
|  | | | |
| Parameters used to evaluate clusters | | | |
| Test | | **Parameter(s) included** | |
| Epidemiological data | |  |  |
|  | | Sports type | 1 = ice hockey  2 = football  3 = ski and snowboard  4 = no sport  5 = handball  6 = automobile  7 = motorcycle  8 = rider  9 = cyclist  10 = others |
|  | | Level of play | 0 = none  1 = elite  2 = amateur |
| Symptoms reported at the start of diagnostic tests | | |  |
|  | | Amnesia anterograde  Amnesia retrograde   - Dizziness - Blurred vision - Headache - Balance problems - Coordination problems - Feeling confused - Difficulty remembering - Nausea - Sensitive to light - Neck pain - Difficulty concentrating   Feeling slowed down | 0 = No  1 = Yes |
