# Supplementary material for: Artificial intelligence for understanding concussion: Retrospective cluster analysis on the balance and vestibular diagnostic data of concussion patients
Source: PLoS One. 2019 Apr 2;14(4):e0214525. doi: 10.1371/journal.pone.0214525 (PMC6445465; doi:10.1371/journal.pone.0214525)
Supplement: S2 Table — (DOCX) [file pone.0214525.s002.docx]

S2 Table PCA Outcomes:

| **Total Variance Explained** | | | | | | | | | |
| --- | --- | --- | --- | --- | --- | --- | --- | --- | --- |
| Component | Initial Eigenvalues | | | Extraction Sums of Squared Loadings | | | Rotation Sums of Squared Loadings | | |
|  | Total | % of Variance | Cumulative % | Total | % of Variance | Cumulative % | Total | % of Variance | Cumulative % |
| 1 | 6.753 | 12.741 | 12.741 | 6.753 | 12.741 | 12.741 | 5.833 | 11.006 | 11.006 |
| 2 | 5.595 | 10.557 | 23.299 | 5.595 | 10.557 | 23.299 | 4.424 | 8.348 | 19.354 |
| 3 | 4.504 | 8.497 | 31.796 | 4.504 | 8.497 | 31.796 | 4.005 | 7.556 | 26.910 |
| 4 | 3.610 | 6.811 | 38.607 | 3.610 | 6.811 | 38.607 | 3.401 | 6.418 | 33.327 |
| 5 | 3.216 | 6.067 | 44.674 | 3.216 | 6.067 | 44.674 | 3.359 | 6.338 | 39.665 |
| 6 | 2.876 | 5.426 | 50.100 | 2.876 | 5.426 | 50.100 | 3.098 | 5.845 | 45.510 |
| 7 | 2.700 | 5.094 | 55.194 | 2.700 | 5.094 | 55.194 | 2.993 | 5.646 | 51.157 |
| 8 | 2.221 | 4.191 | 59.385 | 2.221 | 4.191 | 59.385 | 2.418 | 4.561 | 55.718 |
| 9 | 2.190 | 4.133 | 63.518 | 2.190 | 4.133 | 63.518 | 2.236 | 4.219 | 59.937 |
| 10 | 1.813 | 3.421 | 66.938 | 1.813 | 3.421 | 66.938 | 1.921 | 3.625 | 63.562 |
| 11 | 1.566 | 2.954 | 69.893 | 1.566 | 2.954 | 69.893 | 1.846 | 3.483 | 67.045 |
| 12 | 1.417 | 2.674 | 72.566 | 1.417 | 2.674 | 72.566 | 1.691 | 3.191 | 70.236 |
| 13 | 1.272 | 2.400 | 74.967 | 1.272 | 2.400 | 74.967 | 1.472 | 2.776 | 73.012 |
| 14 | 1.198 | 2.260 | 77.227 | 1.198 | 2.260 | 77.227 | 1.423 | 2.685 | 75.698 |
| 15 | 1.085 | 2.047 | 79.273 | 1.085 | 2.047 | 79.273 | 1.417 | 2.673 | 78.371 |
| 16 | 1.039 | 1.960 | 81.233 | 1.039 | 1.960 | 81.233 | 1.338 | 2.525 | 80.897 |
| 17 | 1.004 | 1.894 | 83.128 | 1.004 | 1.894 | 83.128 | 1.182 | 2.231 | 83.128 |
| 18 | .908 | 1.713 | 84.841 |  |  |  |  |  |  |
| 19 | .800 | 1.509 | 86.350 |  |  |  |  |  |  |
| 20 | .748 | 1.410 | 87.760 |  |  |  |  |  |  |
| 21 | .696 | 1.312 | 89.073 |  |  |  |  |  |  |
| 22 | .669 | 1.263 | 90.336 |  |  |  |  |  |  |
| 23 | .594 | 1.121 | 91.456 |  |  |  |  |  |  |
| 24 | .523 | .986 | 92.442 |  |  |  |  |  |  |
| 25 | .478 | .902 | 93.344 |  |  |  |  |  |  |
| 26 | .399 | .752 | 94.096 |  |  |  |  |  |  |
| 27 | .371 | .700 | 94.796 |  |  |  |  |  |  |
| 28 | .353 | .666 | 95.463 |  |  |  |  |  |  |
| 29 | .327 | .617 | 96.079 |  |  |  |  |  |  |
| 30 | .309 | .583 | 96.662 |  |  |  |  |  |  |
| 31 | .282 | .531 | 97.194 |  |  |  |  |  |  |
| 32 | .232 | .438 | 97.632 |  |  |  |  |  |  |
| 33 | .209 | .394 | 98.025 |  |  |  |  |  |  |
| 34 | .187 | .353 | 98.378 |  |  |  |  |  |  |
| 35 | .156 | .294 | 98.673 |  |  |  |  |  |  |
| 36 | .148 | .279 | 98.951 |  |  |  |  |  |  |
| 37 | .121 | .229 | 99.180 |  |  |  |  |  |  |
| 38 | .107 | .201 | 99.382 |  |  |  |  |  |  |
| 39 | .082 | .154 | 99.536 |  |  |  |  |  |  |
| 40 | .066 | .125 | 99.660 |  |  |  |  |  |  |
| 41 | .056 | .105 | 99.765 |  |  |  |  |  |  |
| 42 | .049 | .093 | 99.858 |  |  |  |  |  |  |
| 43 | .038 | .071 | 99.929 |  |  |  |  |  |  |
| 44 | .021 | .039 | 99.968 |  |  |  |  |  |  |
| 45 | .014 | .027 | 99.995 |  |  |  |  |  |  |
| 46 | .003 | .005 | 100.000 |  |  |  |  |  |  |
| 47 | 5.827E-5 | .000 | 100.000 |  |  |  |  |  |  |
| 48 | 1.346E-5 | 2.539E-5 | 100.000 |  |  |  |  |  |  |
| 49 | 7.734E-6 | 1.459E-5 | 100.000 |  |  |  |  |  |  |
| 50 | 3.751E-6 | 7.077E-6 | 100.000 |  |  |  |  |  |  |
| 51 | 7.298E-10 | 1.377E-9 | 100.000 |  |  |  |  |  |  |
| 52 | 3.209E-10 | 6.055E-10 | 100.000 |  |  |  |  |  |  |
| 53 | -8.590E-18 | -1.621E-17 | 100.000 |  |  |  |  |  |  |

| **Rotated Component Matrix^a^** | | | | | | | | | | | | |
| --- | --- | --- | --- | --- | --- | --- | --- | --- | --- | --- | --- | --- |
|  | Component | | | | | | | | | | | |
|  | 1 | 2 | 3 | 4 | 5 | 6 | 7 | 8 | 9 | 10 | 11 | 12 |
| Zscore(HITpostR) | .969 |  |  |  |  |  |  |  |  |  |  |  |
| Zscore(HITantL) | .969 |  |  |  |  |  |  |  |  |  |  |  |
| Zscore(HITantR) | .943 |  |  |  |  |  |  |  |  |  |  |  |
| Zscore(HITpostAss) | .932 |  |  |  |  |  |  |  |  |  |  |  |
| Zscore(HITantAss) | .932 |  |  |  |  |  |  |  |  |  |  |  |
| Zscore(HITpostL) | .898 |  |  |  |  |  |  |  |  |  |  |  |
| Zscore(VNGmGLPsum) |  | .975 |  |  |  |  |  |  |  |  |  |  |
| Zscore(VNGmGLPL30) |  | -.868 |  |  |  |  |  |  |  |  |  |  |
| Zscore(VNGmGLPL44) |  | .866 |  |  |  |  |  |  |  |  |  |  |
| Zscore(VNGmGLPR30) |  | .863 |  |  |  |  |  |  |  |  |  |  |
| Zscore(VNGmGLPR44) |  | -.862 |  |  |  |  |  |  |  |  |  |  |
| Zscore(SOTsum) |  |  | .949 |  |  |  |  |  |  |  |  |  |
| Zscore(SOTvis) |  |  | .881 |  |  |  |  |  |  |  |  |  |
| Zscore(SOTvest) |  |  | .857 |  |  |  |  |  |  |  |  |  |
| Zscore(SOTsom) |  |  | .739 |  |  |  |  |  |  |  |  |  |
| Zscore(SOTpref) |  |  | .600 |  |  |  |  |  |  |  |  |  |
| Sex |  |  | .499 |  |  |  |  |  |  |  |  |  |
| Zscore(DVAstaticL) |  |  |  | .955 |  |  |  |  |  |  |  |  |
| Zscore(DVAstaticR) |  |  |  | .955 |  |  |  |  |  |  |  |  |
| Zscore(DVAdynamicR) |  |  |  | .778 |  | .365 |  |  |  |  |  |  |
| Zscore(DVAdynamicL) |  |  |  | .775 |  | .361 |  |  |  |  |  |  |
| Zscore(VNGindexR44) |  |  |  |  | .899 |  |  |  |  |  |  |  |
| Zscore(VNGindexR30) |  |  |  |  | .832 |  |  |  |  |  |  |  |
| Zscore(SVVleftDegrees) |  |  |  |  | .789 |  |  |  |  |  |  |  |
| Zscore(VNGdiff) |  |  |  |  | .683 |  |  |  |  |  |  |  |
| Zscore: age |  |  |  |  | .545 |  |  |  |  |  |  |  |
| Zscore(DVAvL) |  |  |  |  |  | .898 |  |  |  |  |  |  |
| Zscore(DVAvR) |  |  |  |  |  | .875 |  |  |  |  |  |  |
| Zscore(DVAverlustR) |  |  |  |  |  | .672 |  |  |  |  |  |  |
| Zscore(DVAverlustL) |  |  |  |  |  | .670 |  |  |  |  |  |  |
| Zscore(HITlatL) |  |  |  |  |  |  | .955 |  |  |  |  |  |
| Zscore(HITlatR) |  |  |  |  |  |  | .955 |  |  |  |  |  |
| Zscore(HITlatAss) |  |  |  |  |  |  | .835 |  |  |  |  |  |
| Zscore(SVVrightDegrees) |  |  |  |  |  |  | .546 |  |  |  |  |  |
| Zscore(SVVoverallMean) |  |  |  |  |  |  |  | .817 |  |  |  |  |
| Zscore(SVVstraitSD) |  |  |  |  |  |  |  | .769 |  | .303 |  |  |
| Zscore(SVVstraitMean) |  |  |  |  |  |  |  | .687 |  |  |  |  |
| Zscore(FUNDUSdiff) |  |  |  |  |  |  |  |  | .864 |  |  |  |
| Zscore(FUNDUSleft) |  |  |  |  |  |  |  |  | -.821 |  |  |  |
| Zscore(SVVstraitDegrees) |  |  |  |  |  |  |  |  | .596 |  |  |  |
| Zscore(SVVleftSD) |  |  |  |  |  |  |  |  |  | .823 |  |  |
| Zscore(SVVoverallSD) |  |  |  |  |  |  |  |  |  | .727 |  | -.303 |
| Zscore(VNGindexL44) |  |  |  |  |  |  |  |  |  |  | .868 |  |
| Zscore(VNGindexL30) |  |  |  |  |  |  |  |  |  |  | .837 |  |
| Zscore(SVVrightMean) |  |  |  |  |  |  |  |  |  |  |  | .800 |
| Zscore(SVVleftMean) |  |  |  |  |  |  |  | .541 |  |  |  | -.558 |
| Zscore(SVVrightSD) |  |  |  |  |  |  |  |  |  |  |  |  |
| Zscore(FUNDUSright) |  |  |  |  |  |  |  |  | .384 |  |  |  |
| Zscore(cVEMPthres) |  |  |  |  |  |  |  |  |  |  |  |  |
| Zscore(TimeDelay) |  |  |  |  |  |  |  |  |  |  |  |  |
| Zscore(VNGdominancy) |  |  |  |  |  |  |  |  |  |  |  |  |
| Zscore(oVEMP) |  |  |  |  |  |  |  |  |  |  |  |  |
| Zscore(cVEMPass) |  |  |  |  |  |  |  |  |  |  |  |  |
