# Supplementary figures and images for: Artificial intelligence for understanding concussion: Retrospective cluster analysis on the balance and vestibular diagnostic data of concussion patients
Source: PLoS One. 2019 Apr 2;14(4):e0214525. doi: 10.1371/journal.pone.0214525 (PMC6445465; doi:10.1371/journal.pone.0214525)

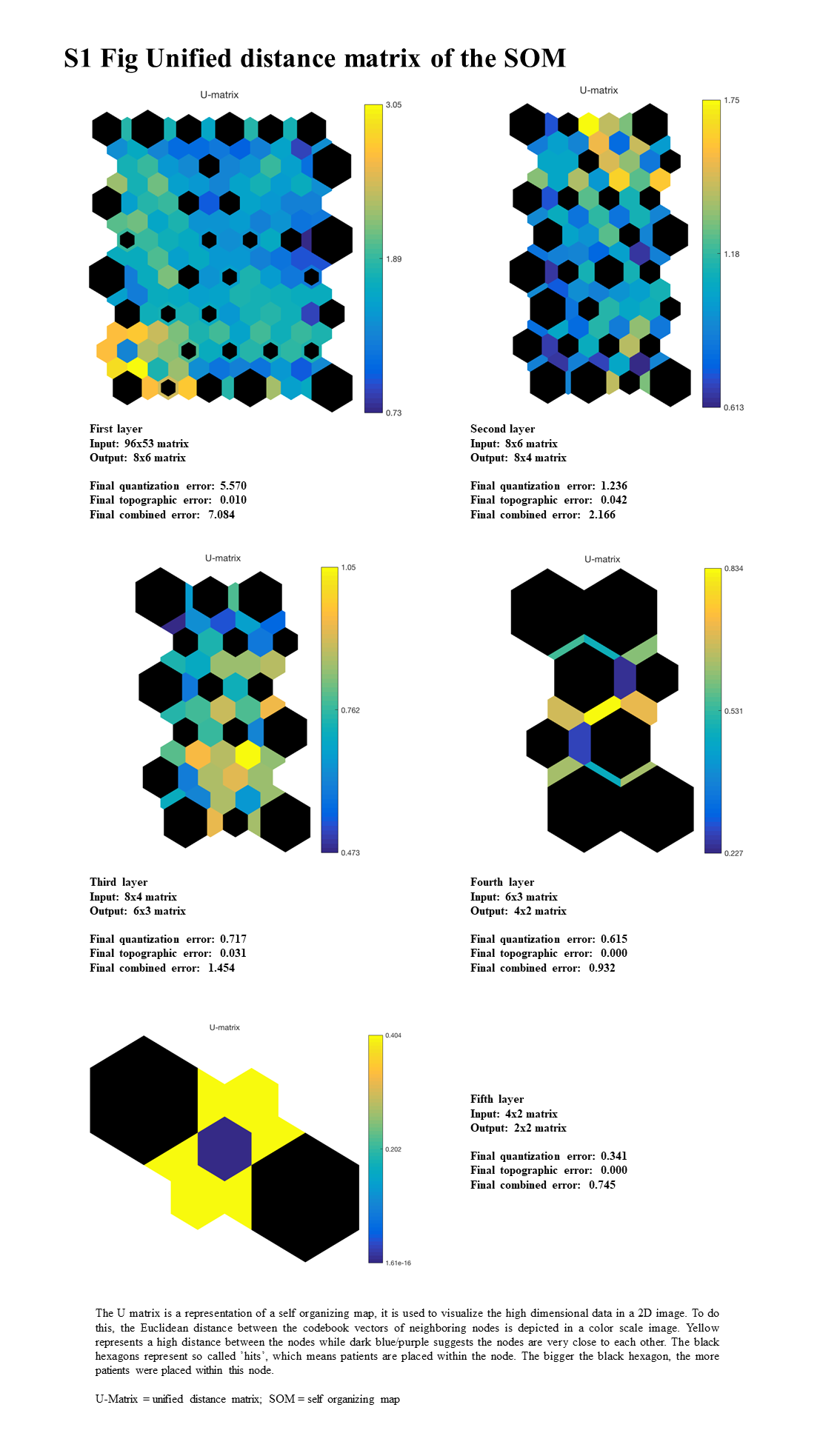

Supplement: S1 Fig — (TIF) [file pone.0214525.s003.tif]

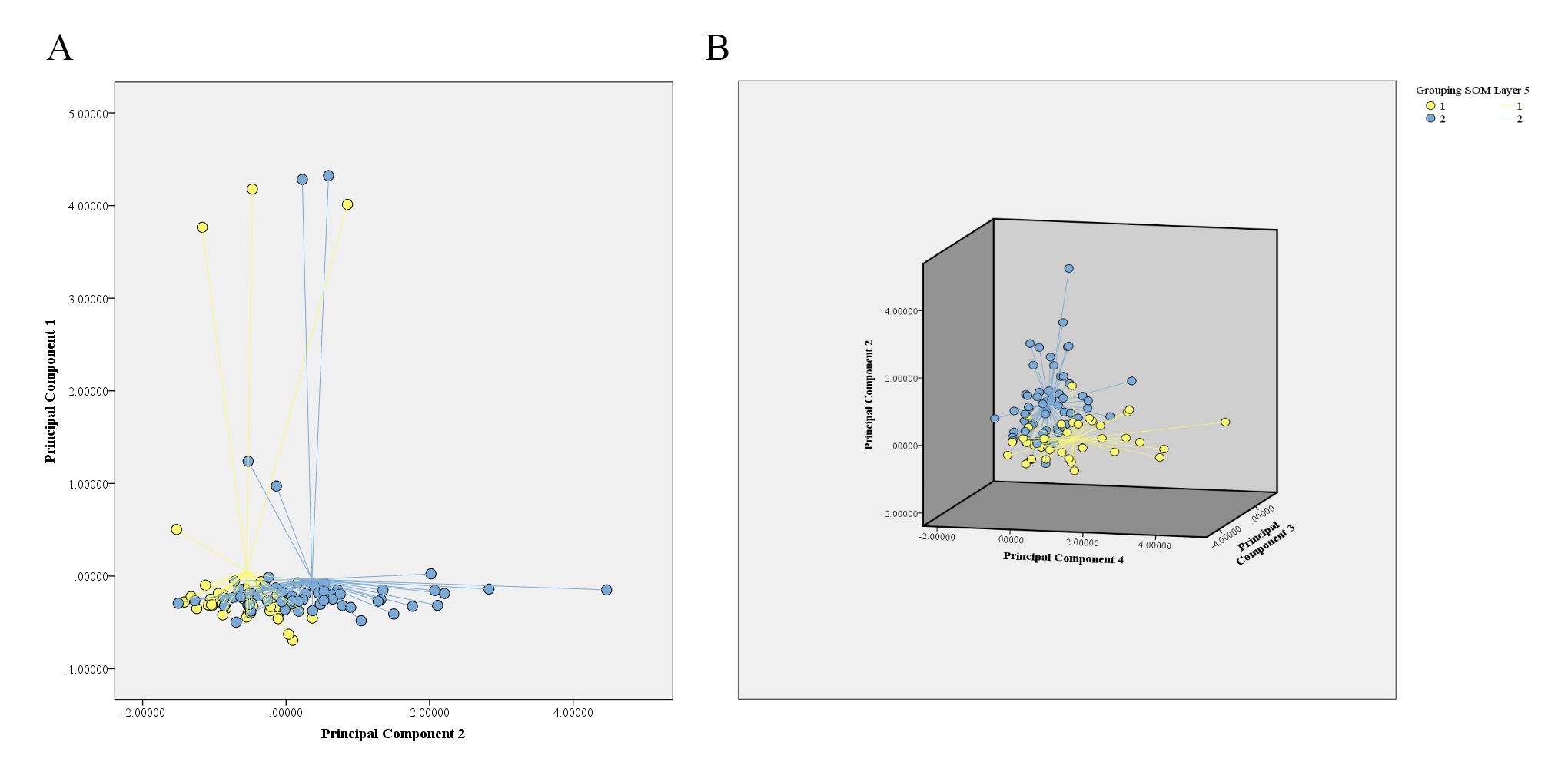

Supplement: S2 Fig — (A) 2D scatter plot: PC 1 plotted against PC 2. (B) 3D scatter plot: PC 2, 3, and 4. Yellow markers were clustered in group-1; Blue markers were clustered in group-2. (TIF) [file pone.0214525.s004.tif]
